# Supplementary material for: Geographic potential of the world’s largest hornet, Vespa mandarinia Smith (Hymenoptera: Vespidae), worldwide and particularly in North America
Source: PeerJ. 2021 Jan 13;9:e10690. doi: 10.7717/peerj.10690 (PMC7811286; doi:10.7717/peerj.10690)
Supplement: Supplemental Information 1 — BIO3: isothermality, BIO5: maximum temperature of warmest month, BIO6: minimum temperature of coldest month, BIO7: temperature annual range, BIO13: specific humidity of most humid month, BIO14: specific humidity of least humid month. [file peerj-09-10690-s001.docx]

Table S1. Sets of environmental predictors obtained with all combinations of two or more raw variables (BIO3: isothermality, BIO5: maximum temperature of warmest month, BIO6: minimum temperature of coldest month, BIO7: temperature annual range, BIO13: specific humidity of most humid month, BIO14: specific humidity of least humid month).

| Set name | BIO3 | BIO5 | BIO6 | BIO7 | BIO13 | BIO14 |
| --- | --- | --- | --- | --- | --- | --- |
| Set 1 |  |  |  |  | X | X |
| Set 2 | X |  |  |  | X |  |
| Set 3 |  | X |  |  | X |  |
| Set 4 |  |  | X |  | X |  |
| Set 5 |  |  |  | X | X |  |
| Set 6 |  |  | X |  |  | X |
| Set 7 |  | X |  |  |  | X |
| Set 8 |  |  | X |  |  | X |
| Set 9 |  |  |  | X |  | X |
| Set 10 | X | X |  |  |  |  |
| Set 11 | X |  | X |  |  |  |
| Set 12 | X |  |  | X |  |  |
| Set 13 |  | X | X |  |  |  |
| Set 14 |  | X |  | X |  |  |
| Set 15 |  |  | X | X |  |  |
| Set 16 | X |  |  |  | X | X |
| Set 17 |  | X |  |  | X | X |
| Set 18 |  |  | X |  | X | X |
| Set 19 |  |  |  | X | X | X |
| Set 20 | X | X |  |  | X |  |
| Set 21 | X |  | X |  | X |  |
| Set 22 | X |  |  | X | X |  |
| Set 23 |  | X | X |  | X |  |
| Set 24 |  | X |  | X | X |  |
| Set 25 |  |  | X | X | X |  |
| Set 26 | X | X |  |  |  | X |
| Set 27 | X |  | X |  |  | X |
| Set 28 | X |  |  | X |  | X |
| Set 29 |  | X | X |  |  | X |
| Set 30 |  | X |  | X |  | X |
| Set 31 |  |  | X | X |  | X |
| Set 32 | X | X | X |  |  |  |
| Set 33 | X | X |  | X |  |  |
| Set 34 | X |  | X | X |  |  |
| Set 35 |  | X | X | X |  |  |
| Set 36 | X | X |  |  | X | X |
| Set 37 | X |  | X |  | X | X |
| Set 38 | X |  |  | X | X | X |
| Set 39 |  | X | X |  | X | X |
| Set 40 |  | X |  | X | X | X |
| Set 41 |  |  | X | X | X | X |
| Set 42 | X | X | X |  | X |  |
| Set 43 | X | X |  | X | X |  |
| Set 44 | X |  | X | X | X |  |
| Set 45 |  | X | X | X | X |  |
| Set 46 | X | X | X |  |  | X |
| Set 47 | X | X |  | X |  | X |
| Set 48 | X |  | X | X |  | X |
| Set 49 |  | X | X | X |  | X |
| Set 50 | X | X | X | X |  |  |
| Set 51 | X | X | X |  | X | X |
| Set 52 | X | X |  | X | X | X |
| Set 53 | X |  | X | X | X | X |
| Set 54 |  | X | X | X | X | X |
| Set 55 | X | X | X | X | X |  |
| Set 56 | X | X | X | X |  | X |
| Set 57 | X | X | X | X | X | X |
