# Supplementary material for: Geographic potential of the world’s largest hornet, Vespa mandarinia Smith (Hymenoptera: Vespidae), worldwide and particularly in North America
Source: PeerJ. 2021 Jan 13;9:e10690. doi: 10.7717/peerj.10690 (PMC7811286; doi:10.7717/peerj.10690)
Supplement: Supplemental Information 2 [file peerj-09-10690-s002.docx]

Table S2. Sets of environmental predictors obtained with all combinations of two or more principal components (PCs).

| Set name | PC1 | PC2 | PC3 | PC4 |
| --- | --- | --- | --- | --- |
| Set 1 | X | X |  |  |
| Set 2 | X |  | X |  |
| Set 3 | X |  |  | X |
| Set 4 |  | X | X |  |
| Set 5 |  | X |  | X |
| Set 6 |  |  | X | X |
| Set 7 | X | X | X |  |
| Set 8 | X | X |  | X |
| Set 9 | X |  | X | X |
| Set 10 |  | X | X | X |
| Set 11 | X | X | X | X |
