# Supplementary material for: Geographic potential of the world’s largest hornet, Vespa mandarinia Smith (Hymenoptera: Vespidae), worldwide and particularly in North America
Source: PeerJ. 2021 Jan 13;9:e10690. doi: 10.7717/peerj.10690 (PMC7811286; doi:10.7717/peerj.10690)
Supplement: Supplemental Information 3 [file peerj-09-10690-s003.docx]

Table S3. Proportion of suitable areas obtained for *Vespa mandarinia* in North America, before and after trimming the models with their MOP.

| MOP trimming | Model scheme | Total area (km^2^) | Area predicted as suitable (km^2^) | Proportion of suitable areas |
| --- | --- | --- | --- | --- |
| No trimming | Raw variables and distance thinned occurrences | 20965151.288 | 3839474.074 | 0.183 |
|  | PCs and distance thinned occurrences | 20965151.288 | 6453843.348 | 0.308 |
|  | Raw variables and country-density thinned occurrences | 20965151.288 | 3774173.917 | 0.180 |
|  | PCs and country-density thinned occurrences | 20965151.288 | 6863510.138 | 0.327 |
| After trimming | Raw variables and distance thinned occurrences | 20965151.288 | 3580694.551 | 0.171 |
|  | PCs and distance thinned occurrences | 20965151.288 | 5201290.428 | 0.248 |
|  | Raw variables and country-density thinned occurrences | 20965151.288 | 3428581.505 | 0.164 |
|  | PCs and country-density thinned occurrences | 20965151.288 | 5006070.31 | 0.239 |
