# Supplementary figures and images for: Geographic potential of the world’s largest hornet, Vespa mandarinia Smith (Hymenoptera: Vespidae), worldwide and particularly in North America
Source: PeerJ. 2021 Jan 13;9:e10690. doi: 10.7717/peerj.10690 (PMC7811286; doi:10.7717/peerj.10690)

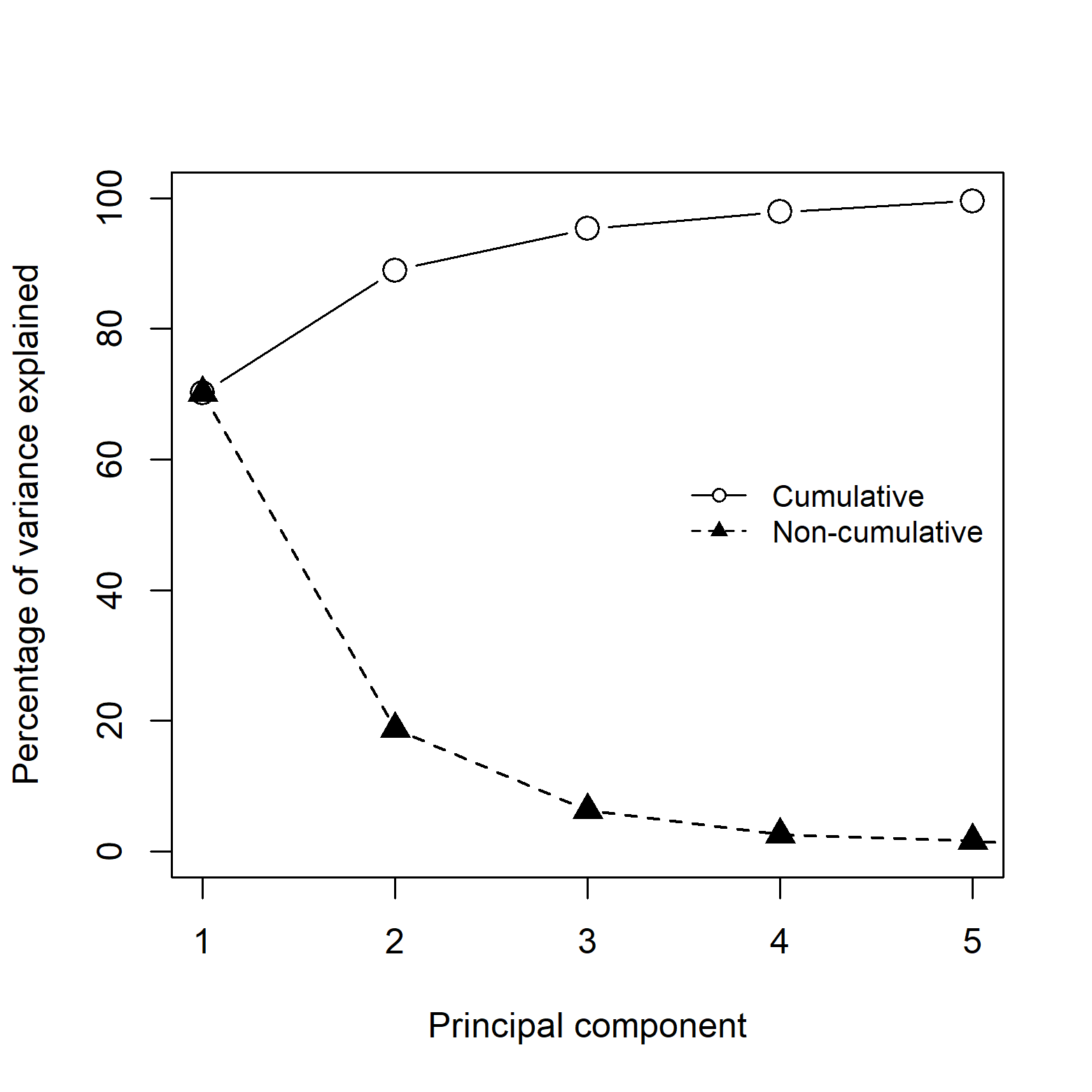

Supplement: Supplemental Information 4 [file peerj-09-10690-s004.png]

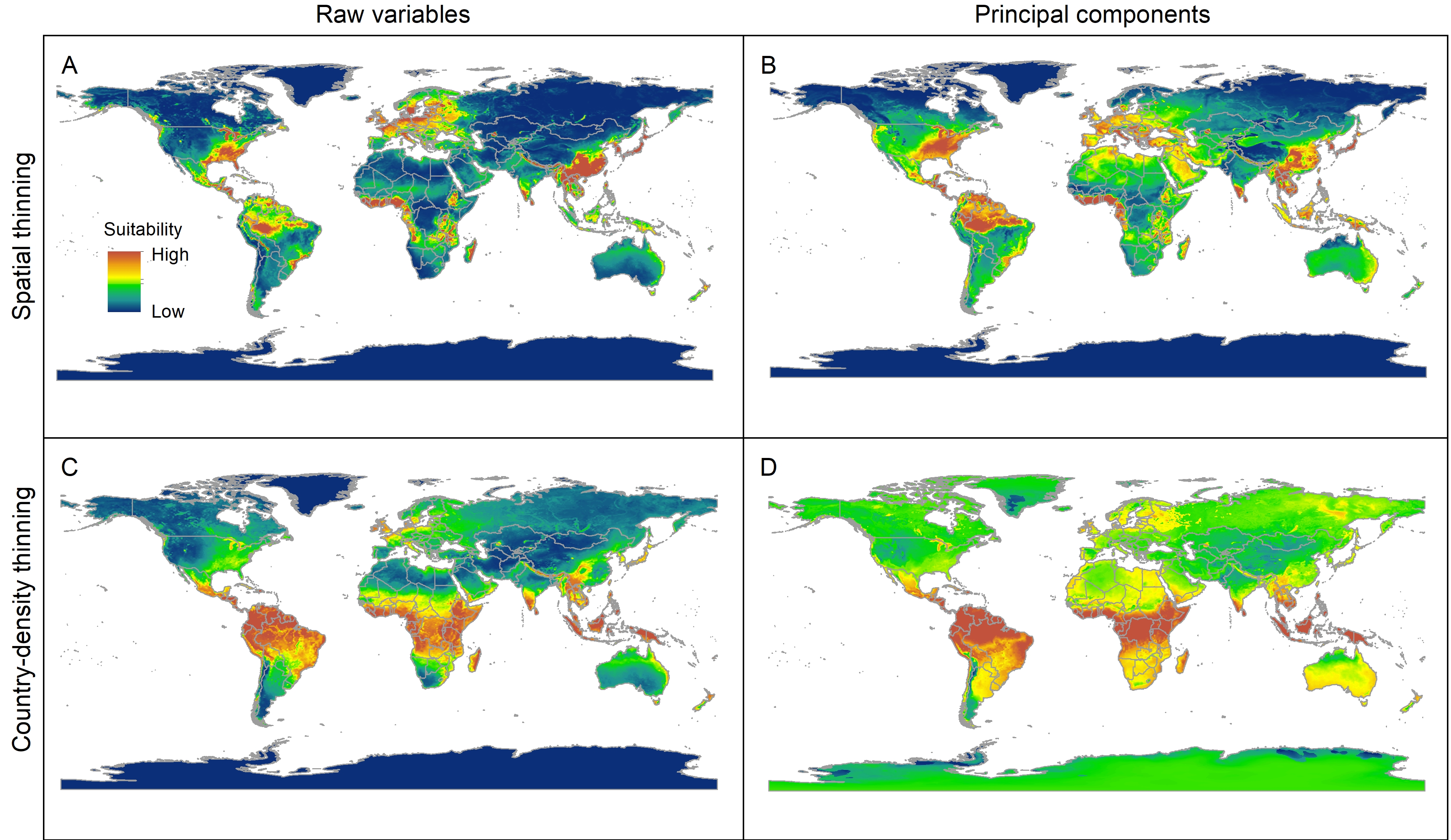

Supplement: Supplemental Information 5 [file peerj-09-10690-s005.png]

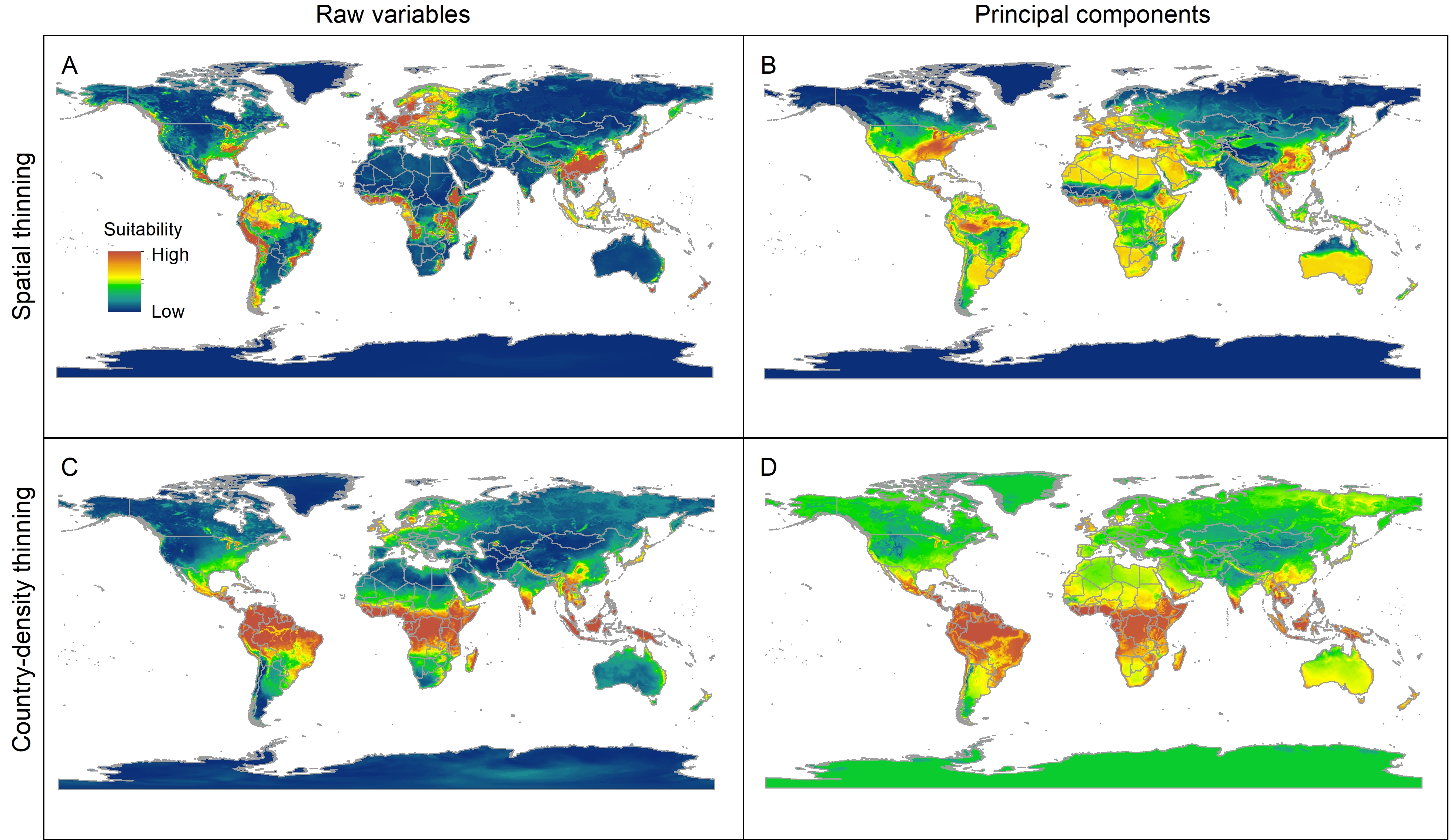

Supplement: Supplemental Information 6 [file peerj-09-10690-s006.png]

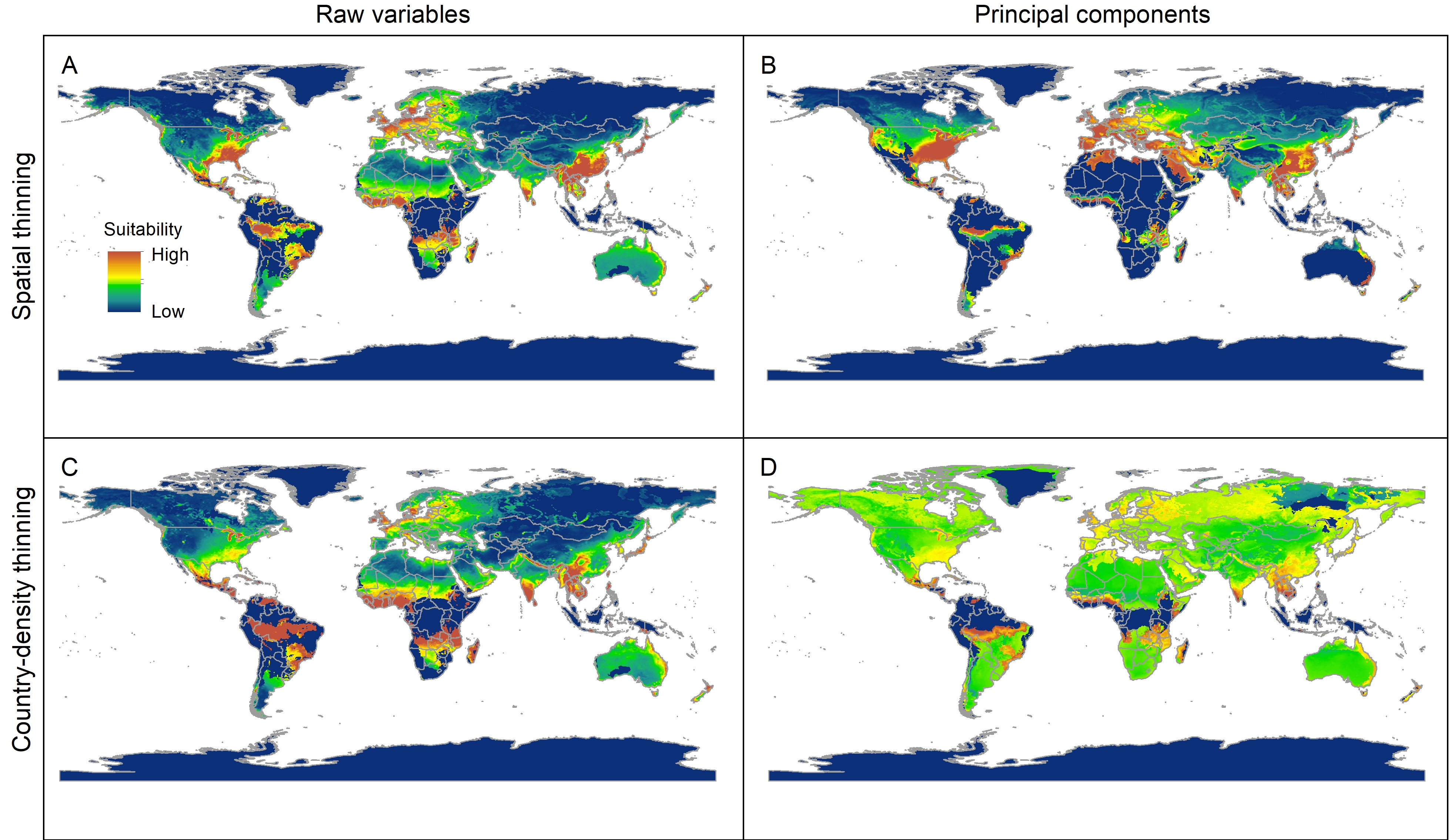

Supplement: Supplemental Information 7 [file peerj-09-10690-s007.png]

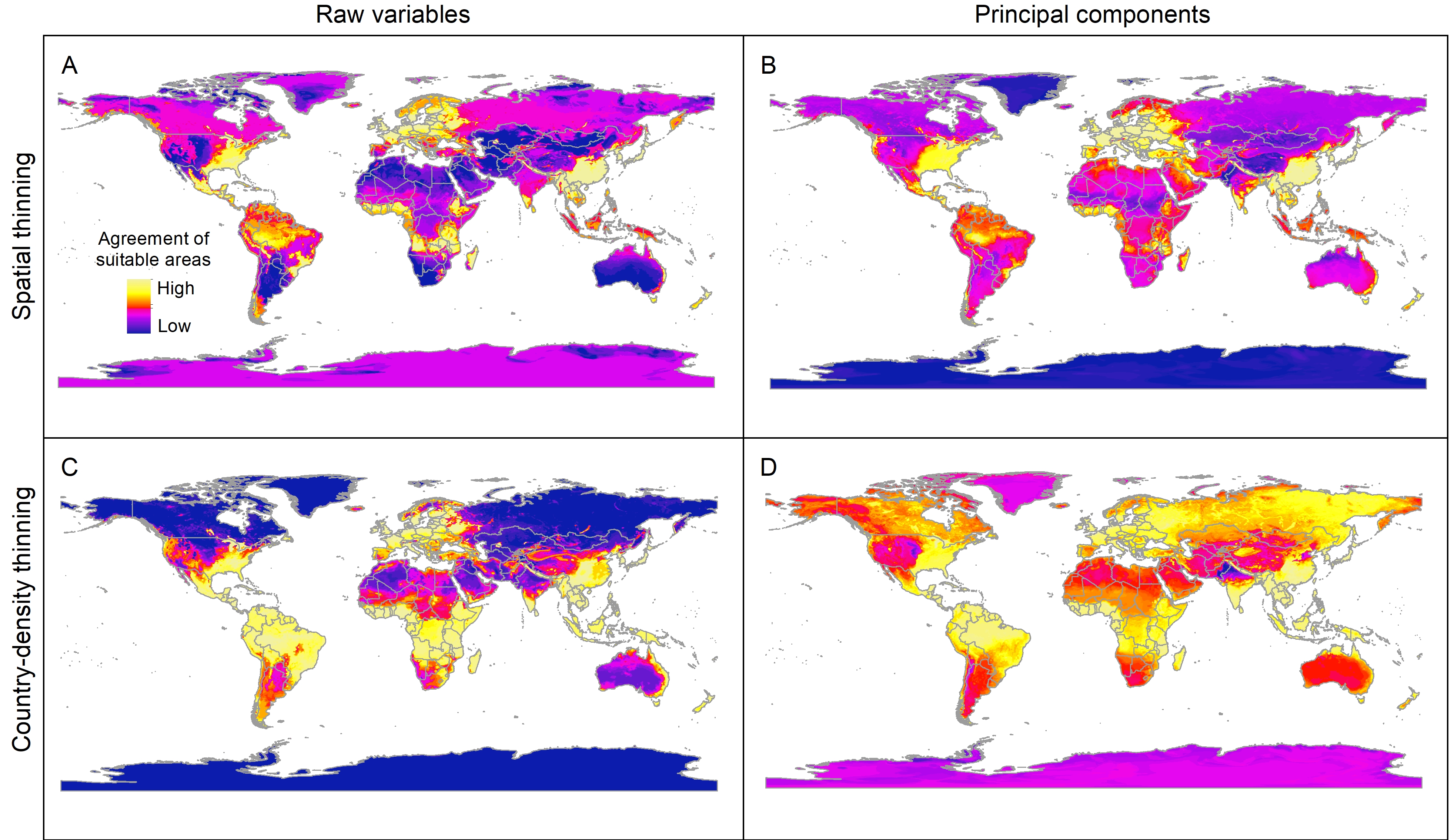

Supplement: Supplemental Information 8 [file peerj-09-10690-s008.png]

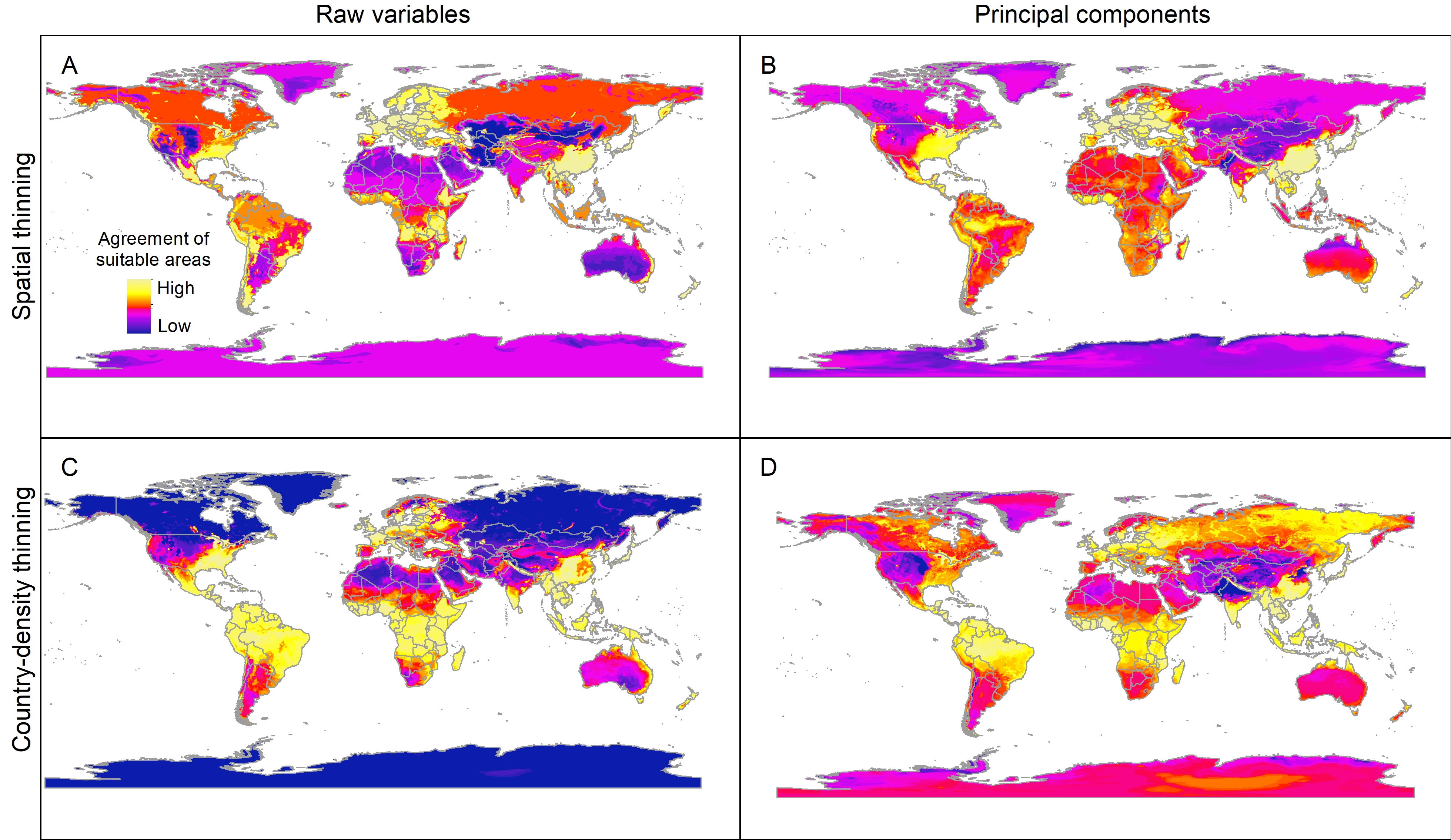

Supplement: Supplemental Information 9 [file peerj-09-10690-s009.png]

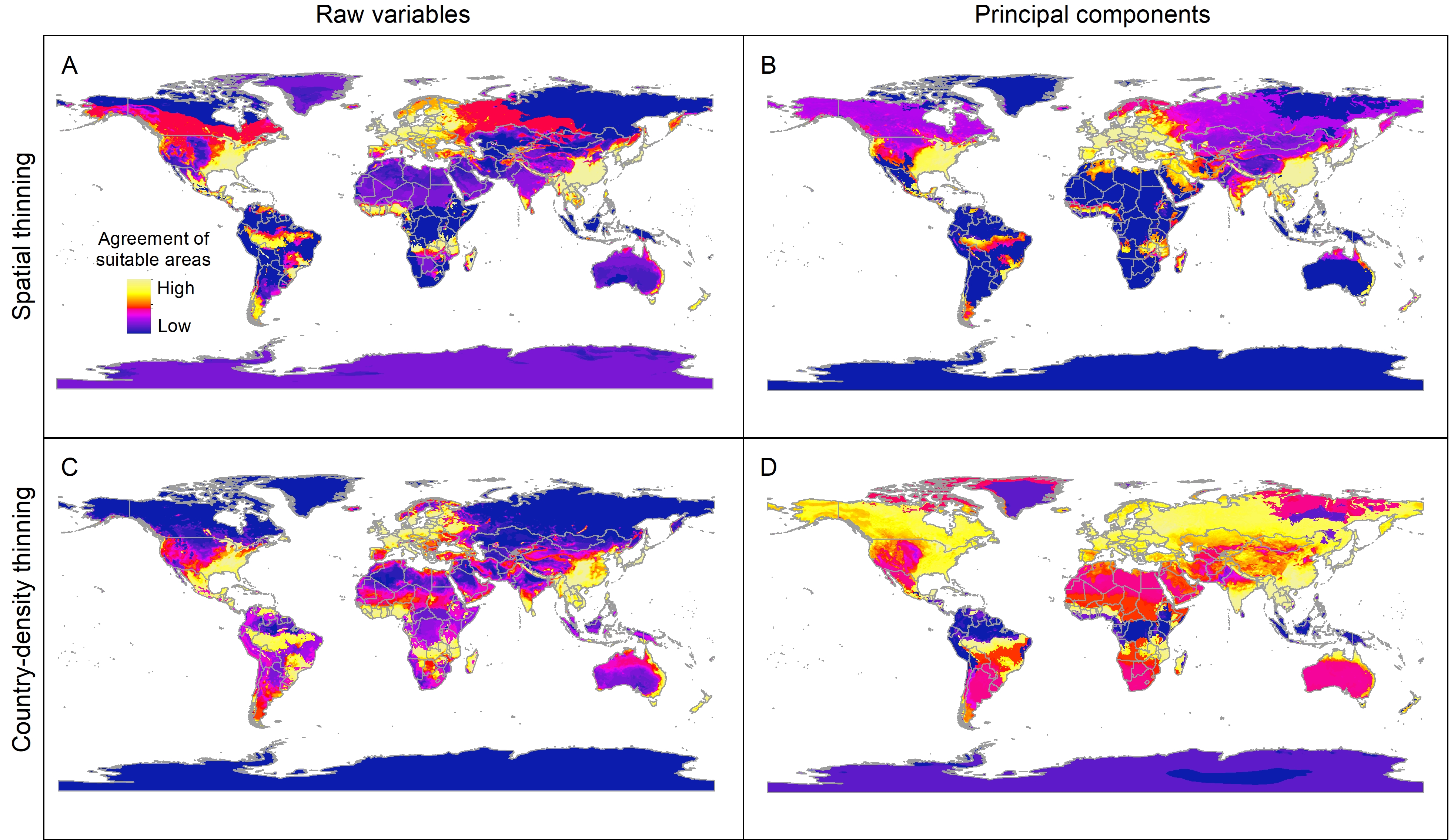

Supplement: Supplemental Information 10 [file peerj-09-10690-s010.png]

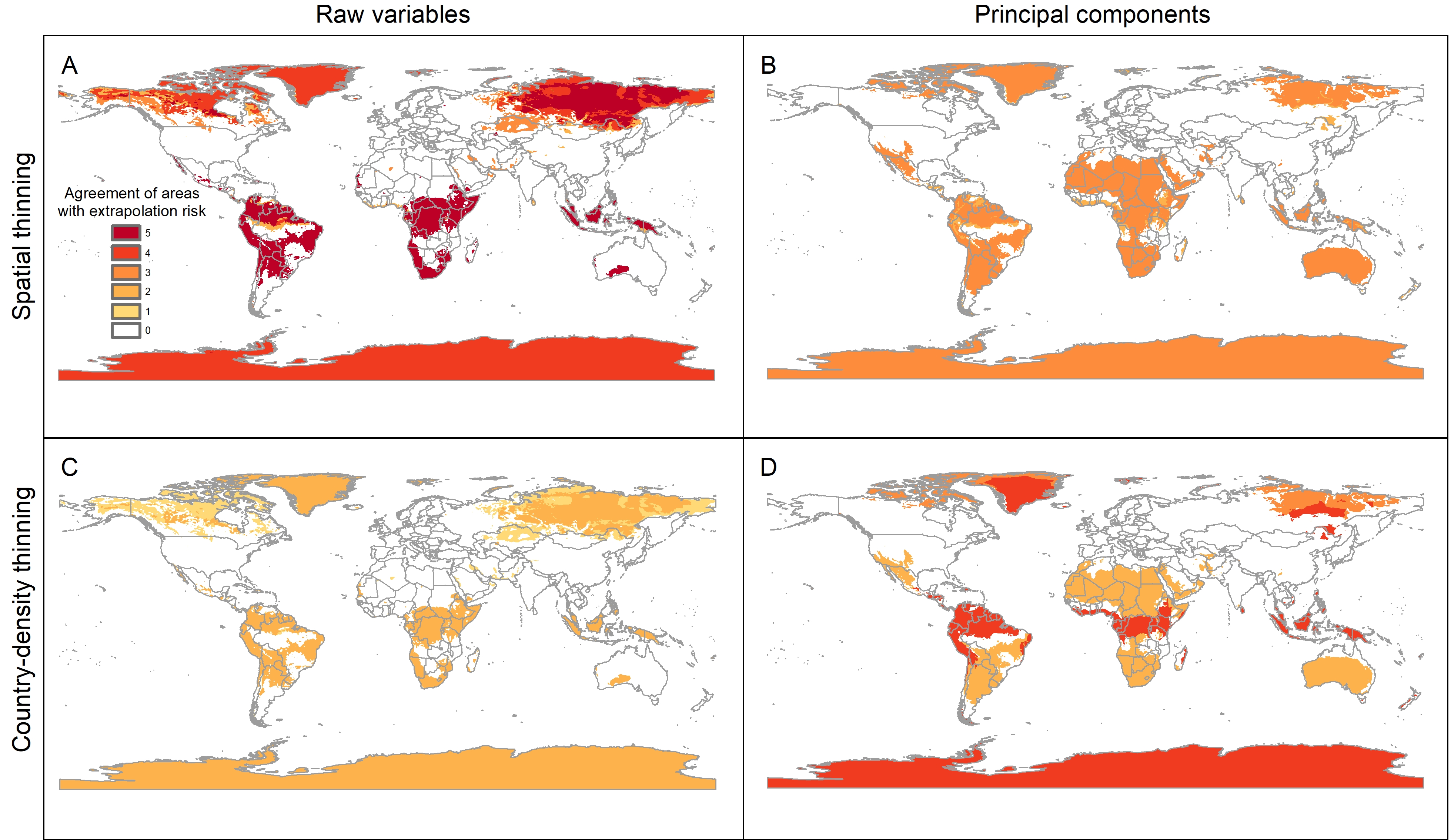

Supplement: Supplemental Information 11 [file peerj-09-10690-s011.png]
