# Supplementary material for: Geographic potential of the world’s largest hornet, Vespa mandarinia Smith (Hymenoptera: Vespidae), worldwide and particularly in North America
Source: PeerJ. 2021 Jan 13;9:e10690. doi: 10.7717/peerj.10690 (PMC7811286; doi:10.7717/peerj.10690)

**Simulations of the likely invasion dynamics of *Vespa mandarinia***

Using the known invaded localities of the AGH in North America as a starting point, we performed simulations of the potential invasion dynamics of this species. All simulations were done in R using the bam package (https://github.com/luismurao/bam), which is based on a cellular automaton discrete model and simulates how species distributions change as a function of the dispersal ability of the species, given an ecological niche model. Here, we describe the steps we followed to create the different invasion scenarios.

To obtain the occupied area at time *t +1*, for each of the schemes considered to obtain ecological niche models for the species, the algorithm calculates the product of three matrices: (i) the suitability matrix for the species (also called the binary map), (ii) the connectivity matrix that defines the connected patches where the species has the ability to reach in one unit of time, and (iii) the matrix of occupied area at time T. Besides considering the four schemes used to obtain the ecological niche models, we created different invasion scenarios by varying (1) the dispersal parameter, *d*, that determines how far the species is able to move in one unit of time; and (2) the suitability threshold used to create the binary maps. The box below describes the algorithm we used to generate simulations for all scenarios.

Notice that we are using ten different values of dispersal and ten different suitability thresholds (steps 1 and 2, respectively). Therefore, after finishing Step 4, we end up with 100 connectivity maps which will be then added together in step 5 to generate the final distribution map under each scheme. Given that the connectivity maps only indicate whether a cell is occupied or not (i.e., they contain only zeros and ones), adding all the connectivity maps together results in cell values from 0 to 100, where 0 indicates that the corresponding cell was never reached by the species, and a value of 100 indicates that 100 of the times the species was able to occupy that cell. Finally, these values can be also used to visualize the dynamics of the invasion through time. We created an animated map by varying the dispersal parameter, d, where we can see the order in which the different cells are reached by the species, indicating the route of potential invasion of *V. mandarinia*.


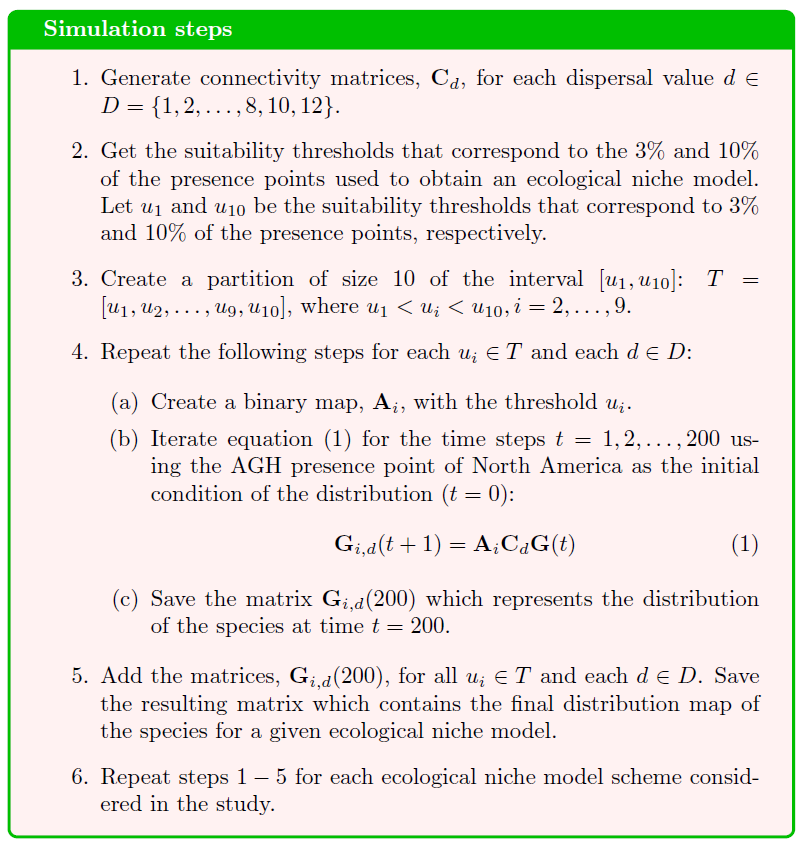

Supplement: Supplemental Information 12 [file peerj-09-10690-s012.docx]
